# Supplementary material for: Chromothripsis during telomere crisis is independent of NHEJ, and consistent with a replicative origin
Source: Genome Res. 2019 May;29(5):737–49. doi: 10.1101/gr.240705.118 (PMC6499312; doi:10.1101/gr.240705.118)
Supplement: Supplemental Material [file supp_gr.240705.118_Supplemental_file_1.zip › contigs/annotated_contigs/DB78/contig.2.DB78_length_405_mean_cov_7.77777777778.docx]

**DB78_length_405_mean_cov_7.77777777778**

```
TGCACAGTGTACTGAATGATTAAGGTAGGTGCTTTCTTATTTTCCTGTCAAGAACATTTATATATCTGGCTACAAAAGTGATATAGGAT
 >chr11:37074484-37074750 + E=9e-149
TACTGACAAAGTTAAAACACACAGAGGGATACAAGACAAAAAACAAAATTGCTTACAACTTTACAAGTCAAAATAACTGTGGCTAACAT

TTTAGTGCATAACTTTAGTGCATTGTTTTTAAAATGACTTTTGTTTTCTTCCATGACCTCTGGGAAGGCCGTTAACTATATATATAT|A

|AACTACCTGAAGAAGGGGAGCAAAGTTGCAGAGGGGATTCAGGTTTTGAAAGGATGGTTAGATTGATGAACTTGGAATCTTTTTCCTC
>chr11:37075941-37076081 - E=9e-73
TGACCAAAAGATTCCATGATTGTATATGTTTATGTAAATATTCTTCTAGTA
```
